# Continuous hemofiltration improves the prognosis of bacterial sepsis complicated by liver dysfunction in children

**DOI:** 10.1186/s12887-018-1243-3

**Published:** 2018-08-11

**Authors:** Yun Cui, Xi Xiong, Fei Wang, Yuqian Ren, Chunxia Wang, Yucai Zhang

**Affiliations:** 1 Department of Critical Care Medicine, Shanghai Children’s Hospital, Shanghai Jiao Tong University, No.355 Luding Road, Putuo District, Shanghai, 200062 China; 2 Institute of Pediatric Critical Care, Shanghai Jiao Tong University, No.355 Luding Road, Putuo District, Shanghai, 200062 China

**Keywords:** Continuous hemofiltration, Bacterial sepsis with liver dysfunction, Mortality, Children

## Abstract

**Background:**

Liver dysfunction is an independent risk factor for poor prognosis of patients with sepsis. The aim of this study is to evaluate the effects of continuous hemofiltration in patients with bacterial sepsis complicated by liver dysfunction.

**Methods:**

We retrospectively analyzed the medical records of 27 cases of bacterial sepsis with liver dysfunction admitted to pediatric intensive care unit (PICU) of Shanghai Children’s Hospital between January 2013 and December 2016.

**Results:**

28-day mortality and length of PICU stay were significantly reduced in the continuous hemofiltration group (*n* = 16) compared with the conventional management group (*n* = 11) (31.3% *vs.* 72.7%, 9 [4–23] *vs.* 14 [4–36], respectively, both *P* < 0.05). The interval time between PICU admission and continuous hemofiltration initiation was (22.06 ± 17.68) h, and the median time of continuous hemofiltration duration was 48 h (31–70 h). After 72 h hemofiltration, the levels of total bilirubin (TBIL), direct bilirubin (DBIL), total bile acids (TBA), ammonia, lactate (Lac), TNF-α and IL-6 were significantly decreased in the continuous hemofiltration group. Moreover, multivariate logistic regression analysis indicated that continuous hemofiltration treatment and the TBIL level were independently associated with 28-day mortality of patients with bacterial sepsis complicated by liver dysfunction.

**Conclusions:**

Continuous hemofiltration significantly decreases the serum levels of TBIL, DBIL, TBA, Lac, ammonia, TNF-α, IL-6, and improves 28-day mortality of patients with bacterial sepsis complicated by liver dysfunction.

**Electronic supplementary material:**

The online version of this article (10.1186/s12887-018-1243-3) contains supplementary material, which is available to authorized users.

## Background

Sepsis is defined as life-threatening organ dysfunction caused by a deregulated host response to infection [1]. Until now, bacteria is still the most common pathogens of sepsis [2]. Specifically, Gram-negative (G-) bacteria are among the most important pathogens of sepsis, and lipopolysaccharide (LPS) is regarded as an important stimulator of triggering the systemic inflammatory reaction [3]. The amount of proinflammatory cytokines including tumor necrosis factor (TNF)-α and interleukin (IL)-6 contribute to liver dysfunction [4]. In addition, the stimulated liver produces and releases high amounts of bioactive lipids and acute phase proteins [5], which also play an important role in sepsis-induced liver dysfunction [6]. Liver dysfunction is an independent risk factor for a poor prognosis of patients with sepsis, and the mortality of sepsis-associated liver dysfunction is 54.3–67.6% [7, 8]. The bacterial sepsis complicated by liver dysfunction in children remains the leading causes of death in pediatric intensive care unit (PICU).

Continuous renal replacement treatment (CRRT) preferred as adjuvant therapy in critically ill patients to improve hemodynamics and fluid balance and remove noxious molecules and cytokines [9–11]. Recently, a retrospective cohort study indicated that continuous hemofiltration provides stability and bridge to liver transplantation in patients with pediatric acute liver failure [12]. However, there is little study available about the continuous hemofiltration in sepsis complicated by liver dysfunction, especially initially infected by bacteria in children. In the present study, we retrospectively analyzed the medical records of patients with bacterial sepsis complicated by liver dysfunction admitted to PICU at Shanghai Children’s Hospital from January 2013 to December 2016. The aim of the study was to evaluate the effects of continuous hemofiltration on 28-day mortality and the levels of total bilirubin (TBIL), direct bilirubin (DBIL), total bile acids (TBA), lactate (Lac), ammonia, TNF-α and IL-6 in patients with bacterial sepsis complicated by liver dysfunction.

## Methods

### Study design

We performed a retrospective observational study of patients with bacteria sepsis-associated liver dysfunction admitted to PICU at Shanghai Children’s Hospital between January 2013 and December 2016. The study was conducted in accordance with the ethical principles of the Declaration of Helsinki (and subsequent revisions) and to the current norm for observational studies. This study was approved by the Ethics Committee of Shanghai Children’s Hospital (No. 2016R010-E02). The written informed consents were obtained from all patients’ parents.

### Patients and treatment

Patients more than 1 month and under 14 years old who were diagnosed with sepsis-associated liver dysfunction initially infected by bacteria were screened for inclusion. The patient was diagnosed with sepsis based on the International Pediatric Sepsis consensus conference in 2005 [13] and “Surviving Sepsis Campaign” international guidelines in 2012 [14]. Liver dysfunction is defined as plasma TBIL above 4 mg/dl or 70 μmol/L according to Surviving Sepsis Campaign International Guidelines in 2012 [14]. And patients initially infected by bacteria were confirmed by laboratory finding. Patients discharged 72 h earlier after admission were excluded. And patients with primary hepatobiliary diseases or inherited metabolic diseases were excluded. Primary hepatobiliary involvement was defined as liver trauma, hepatitis, malignancy, and cholecystitis. All patients were treated with conventional management according to “Surviving Sepsis Campaign” international guidelines in 2012 [14]. All the patients’ parents signed the informed consents before continuous hemofilatration treatment if the patients received continuous hemofiltration.

### Continuous hemofiltration

The pattern of CRRT used in patients with bacterial sepsis complicated by liver dysfunction was continuous hemofiltration performed using PRISMA or PRISMA flex blood purification machine and Gambro prisma filter with an ultrafiltrate flow rate of 35–50 mL/kg/hr. The indications for initiation of continuous hemofiltration included acute kidney injury, fluid overload (> 10%), or hyperammonemia (> 100 μmol/L) as described in our previous study [15], as well as hemodynamic instability such as cardiogenic shock, septic shock and multiple organ dysfunction [16, 17]. The patients with severe coagulation disorders (APTT > 80 s or INR > 2.5), or with biofilm allergic reaction, or with difficult to set venin catheter-access were treated with conventional therapies. According to patient body weight, we chose a 6F to 12F central venous catheters (GamCath; Gambro, Colombes, France) to construct the vascular access in the right internal jugular or femoral vein. Continuous hemofiltration was performed as described in our previous study [15]. Briefly, the saline containing 5000–10,000 IU/L heparin was used to pre-treat the filter circuit. And the dosage of heparin was 5-20 U/kg.h to maintain activated partial thromboplastin time (APTT) with 1.5–2 fold of normality during continuous hemofiltration. The replacement solution contains Na ^+^ 130 mmol/L, K ^+^ 4 mmol/L, HCO ^3−^ 28 mmol/L, Ca ^2+^ 1.5 mmol/L, Mg ^2+^ 3.2 mmol/L, Cl ^−^ 109 mmol/L, and glucose 3.7 g/L. The blood flow rate was set as 4–6 mL/kg/min, and the hemofilter was changed for about 24 h or when clotted. When the urine output is over 1 ml/kg.h, fluid overload below 10%, the level of TBIL less than 85 μmol/L or hemodynamics keeping stable, continuous hemofiltration was weaned. Otherwise, continuous hemofiltration should be terminated when children develop severe bleeding or are unable to control acute hemorrhage; or clinical symptoms are not obviously improved after 48 h of treatment.

### Observational data

The demographic data were collected from medical records. The 28-day mortality and the changes of serum levels of biochemical and clinical parameters including alanine aminotransferase (ALT), γ-glutamyltranspeptidase (γ-GT), TBIL, DBIL, TBA, lactate (Lac), ammonia, TNF-α and IL-6, prothrombin time (PT), activated partial thromboplastin time (APTT) and international normalized ratio (INR) were analyzed.

### Statistical analysis

Data were analyzed using SPSS (v.22.0) (SPSS Inc., Chicago, IL). Continuous variables were summarized as means ± standard derivations (SD) for normal distribution data and as median (range) for abnormal distribution data. All variables were tested for normal distribution by using the Kolmogorov-Smirnov test. Partial were transferred to normal distribution by the *Ln* treatment. Student *t* test was used to compare the means of continuous variables and normally distributed data; otherwise, the Mann-Whitney *U* test was used. The *chi*-square test was used to compare the categorical data. The independent factors correlated with 28-day mortality were assessed by applying multivariate logistic regression analysis and Spearman’s rank correlation coefficient. A value of *P* < 0.05 was considered statistically significant.

## Results

**Fig. 1:**
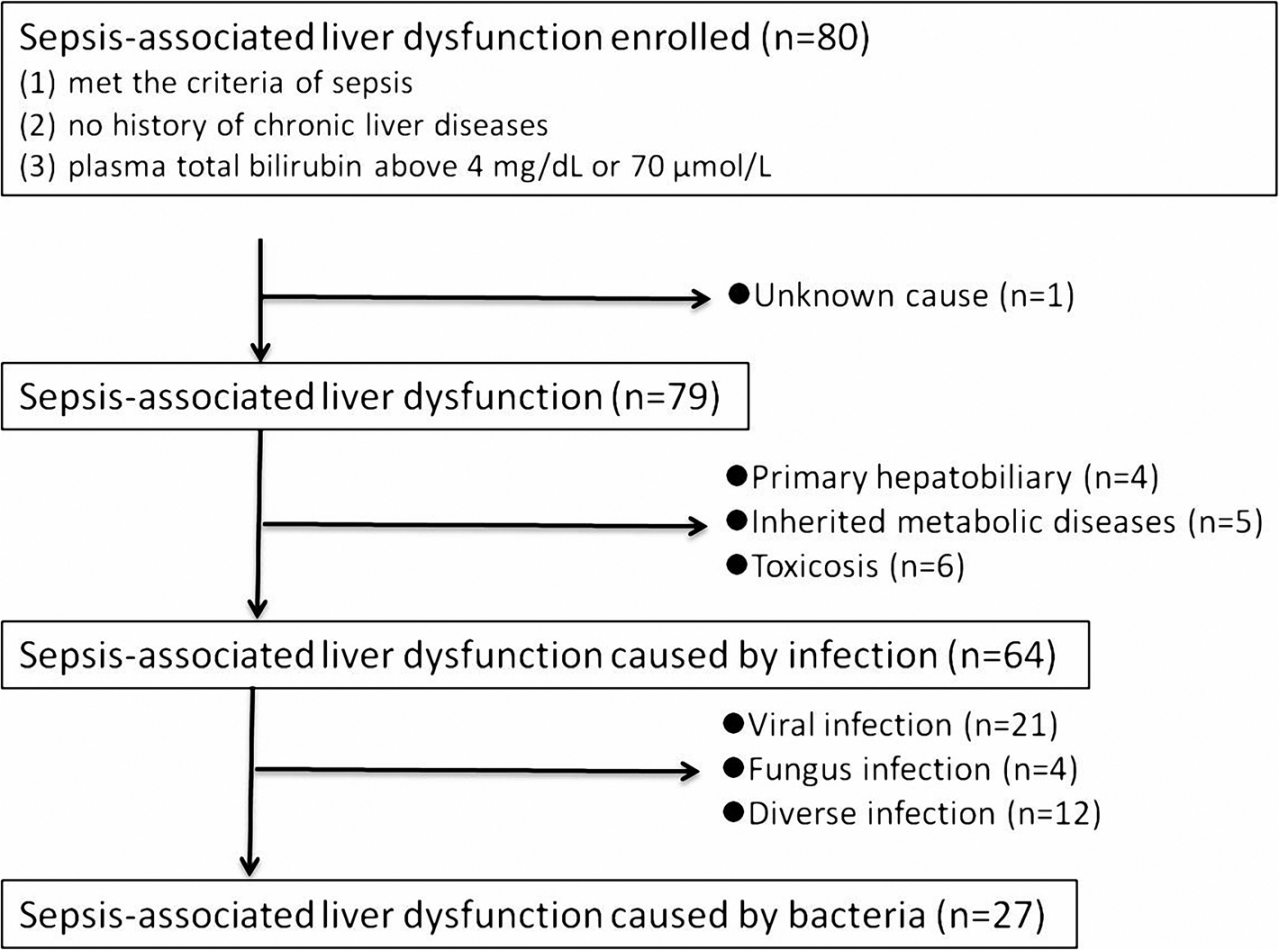
Flowchart of patient enrollment in this study

A total of 80 children diagnosed with sepsis-associated liver dysfunction were admitted to the PICU at Shanghai Children’s Hospital between January 2013 and December 2016. One patient was excluded due to unknown cause. Sixteen patients were excluded due to primary hepatobiliary disease, inherited metabolic diseases and drug intoxication. Thirty-seven patients were excluded due to viral infection, fungal infection or diverse infection. Finally, 27 patients with sepsis-associated liver dysfunction initially infected by bacteria met inclusion criteria, which included 13 male children and 14 female children. And 11 patients received conventional management, and 16 cases were treated with conventional management plus continuous hemofiltration as adjuvant therapy according to medical records (Fig. 1). And the pathogens include *Escherichia coli* (10/27)*, Pseudomonas aeruginosa* (6/27)*, Klebsiella pneumonia* (1/27)*, Streptococcus pneumonia* (7/27) and *Staphylococcus aureus* (3/27). Fourteen patients survived, and 13 patients died. The demographic and clinical characteristics of patients were detailed in Table 1.

**Table 1:** Demographic and clinical characteristics of patients with sepsis complicated by liver dysfunction enrolled in this study

Demographic and Clinical Characteristics	Conventional group (*n* = 11)	Continuous hemofiltration group (*n* = 16)	*P*
Age, month, median (range)	12 (2–192)	54 (3–144)	0.239
Male, n (%)	6 (54.5%)	7 (43.7%)	0.581
Stay in PICU, day, median (range)	14 (4–36)	9 (4–23)	0.022^*^
Body temperature, ^o^C	38.3 (36.0–40.0)	39 (38.0–41.0)	0.080
Hepatomegaly, n (%)	6 (54.5%)	10 (62.5%)	0.679
Hypoproteinemia, n (%)	9 (81.8%)	13 (81.3%)	0.970
Pediatric Risk of Mortality III (PRISM III), median (range)	13(10–18)	14 (10–20)	0.427
Co-organ dysfunction			
ARDS, n (%)	5 (45.4%)	8 (50%)	0.816
Kidney injury, n (%)	4 (36.4%)	7 (43.7%)	0.701
Encephalopathy, n (%)	2 (18.2%)	8 (50%)	0.202
Shock, n (%)	5 (45.4%)	8 (50.0%)	0.816
Infection origin			
Respiratory system, n (%)	5 (45.4%)	6 (37.5%)	0.679
Digestive system, n (%)	3 (27.3%)	4 (25.0%)	0.895
Urinary system, n (%)	1 (9.1%)	2 (12.5%)	0.780
Central nervous system, n (%)	2 (18.2%)	4 (25.0%)	0.673
Pathogens			
*Escherichia coli,* n	4 (9.1%)	6 (37.65%)	0.952
*Pseudomonas aeruginosa,* n	3 (27.3%)	3 (18.8%)	0.603
*Klebsiellapneumoniae,* n	0 (0%)	1 (6.3%)	1.000
*Streptococcus pneumoniae,* n	3 (27.3%)	4 (25.0%)	0.895
*Staphylococcus aureus,* n	1 (9.1%)	2 (12.5%)	0.780
Infection markers			
WBC	11.85 ± 6.90	12.10 ± 8.07	0.933
PCT	5.21 ± 4.29	6.39 ± 4.28	0.489
CRP	98.09 ± 55.29	98.25 ± 53.47	0.994
Liver function			
PT	24.60 ± 22.70	26.78 ± 17.01	0.778
ALT	548.20 ± 264.14	570.00 ± 229.97	0.822
TBIL	176.74 ± 81.01	185.52 ± 142.48	0.855
DBIL	133.34 ± 62.75	141.73 ± 103.95	0.813
28-day mortality, n (%)	8 (72.7%)	5 (31.3%)	0.034^*^

*ARDS* acute respiratory distress syndrome, *MODS* multiple organ dysfunction syndrome, *WBC* white blood cell, ×10^9^/L, *PCT* procalcitonin, ng/mL, *CRP* C-reactive protein, mg/L, *PT* prothrombin time, s, *ALT* alanine transaminase, U/L, *TBIL* total bilirubin, μmol/L, *DBIL* direct bilirubin, μmol/L

* indicates *P* < 0.05 compared with conventional group

### Demographic and clinical characteristics

The characteristics of patients in the conventional management group and the continuous hemofiltration group were similar in respect of age, gender, Pediatric Risk of Mortality III (PRISM III), fever, complications, rate of hepatomegaly or hypoproteinemia, infection origin, pathogens, infection biomarkers and liver function (all *P* > 0.05). The length of PICU stay was significantly shorter in patients of continuous hemofiltration group compared with the conventional management group (9 [4–23] *vs.* 14 [4–36], *P* < 0.05). And 28-day mortality was significantly reduced in patients of continuous hemofiltration group compared with the conventional management group (31.3% *vs. *72.7%, *P* < 0.05, Table 1).

### Clinical benefits of continuous hemofiltration in patients with sepsis-associated liver dysfunction

**Fig. 2:**
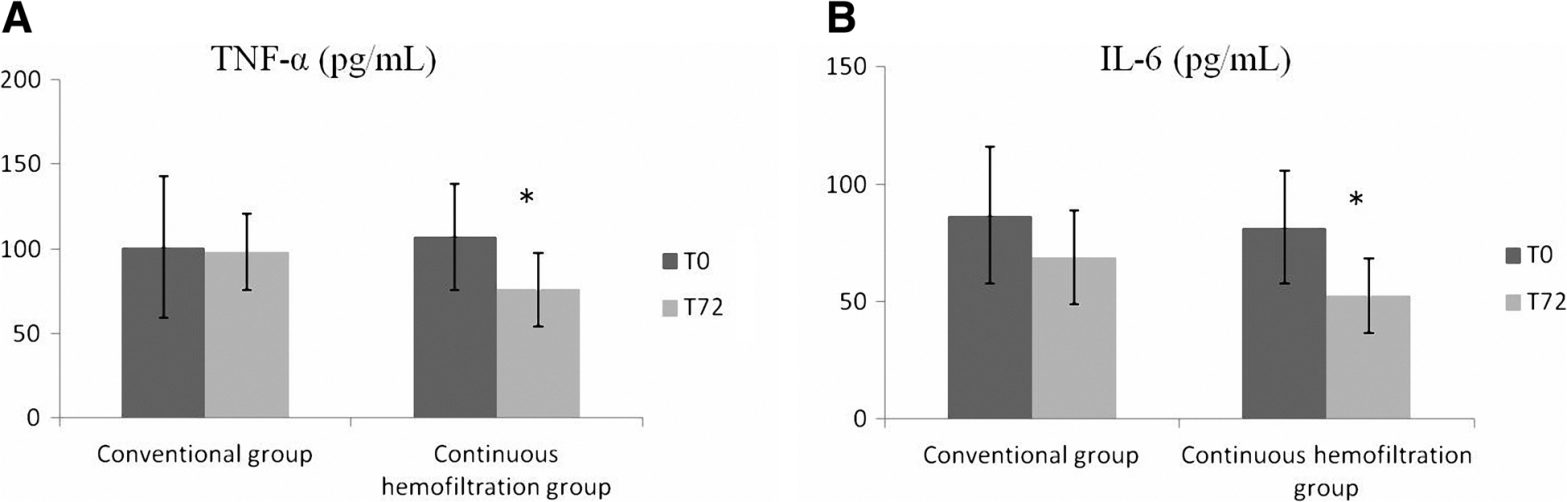
Changes of the serum levels of TNF-α (**a**) and IL-6 (**b**) in patients with sepsis-associated liver dysfunction initially infected by bacteria after treatment with or without continuous hemofiltration. T0: baseline before treatment, T72: 72 h after treatment. * indicates *P* < 0.05 compared with T0

The median interval time between continuous hemofiltration initiation and PICU admission was (22.06 ± 17.68) h. Moreover, the median interval time in non-survivor was significantly longer than that in survivor (37.40 ± 25.53 *vs.* 15.09 ± 6.09, *P* < 0.05). The median time of continuous hemofiltration duration was 48 h (31–70 h) in patients with sepsis-associated liver dysfunction. The 28-day mortality rate in continuous hemofiltration group was 31.3% (5/16), which was significantly decreased compared with the conventional therapy group (72.7%, 8/11). The levels of TBIL, DBIL, TBA, Lac, TNF-α and IL-6 were significantly decreased at 72 h after treatment in the continuous hemofiltration group (all *P* < 0.05), other than in the conventional group (all *P* > 0.05, Table 2). The serum levels of TNF-α and IL-6 decreased about 30–40% in patients treated with continuous hemofiltration during the first 72 h after admission, but not in the conventional group (Fig. 2).

**Table 2:** Changes of the serum levels of biomarkers in patients with sepsis-associated liver dysfunction initially infected by bacteria after treatment with or without continuous hemofiltration

Variables	Conventional group (*n* = 11)			Continuous hemofiltration group (*n* = 16)		
	T0	T72	*P*	T0	T72	*P*
ALT	548.20 ± 264.14	427.55 ± 241.37	0.277	570.00 ± 229.97	418.81 ± 205.74	0.059
AST	1011.11 ± 639.04	721.09 ± 330.22	0.196	1007.81 ± 833.87	611.81 ± 597.51	0.133
γ-GT	185.45 ± 152.1	160.91 ± 126.62	0.685	165.06 ± 83.58	168.15 ± 71.53	0.911
TBIL	176.74 ± 81.01	132.87 ± 75.72	0.204	185.52 ± 142.48	62.59 ± 59.60	0.003^*^
DBIL	133.34 ± 62.75	99.07 ± 43.66	0.153	141.73 ± 103.96	49.05 ± 25.65	0.002^*^
TBA	97.31 ± 33.32	93.95 ± 49.00	0.853	104.73 ± 24.57	78.40 ± 17.54	0.002^*^
ALP	183.27 ± 20.18	175.00 ± 42.37	0.565	159.56 ± 36.35	137.25 ± 59.23	0.209
PT	24.60 ± 22.70	13.53 ± 3.29	0.125	26.78 ± 17.01	14.29 ± 5.89	0.009^*^
APTT	61.56 ± 19.75	53.25 ± 18.04	0.315	63.15 ± 33.76	51.38 ± 22.75	0.256
INR	1.61 ± 0.82	1.48 ± 0.30	0.631	2.20 ± 1.69	1.43 ± 0.36	0.084
Fib	1.57 ± 0.92	2.05 ± 0.70	0.180	1.79 ± 0.64	2.14 ± 0.49	0.097
Lac	5.26 ± 4.48	2.07 ± 1.77	0.040	4.45 ± 3.17	2.33 ± 2.15	0.034^*^
Ammonia	169.44 ± 60.73	109.66 ± 33.35	0.010^*^	153.12 ± 79.66	67.44 ± 26.42	< 0.001^*^
TNF-α	107.01 ± 41.78	98.23 ± 22.95	0.548	107.00 ± 31.35	76.03 ± 21.84	0.003^*^
IL-6	86.75 ± 29.14	69.10 ± 20.00	0.113	81.64 ± 23.85	52.66 ± 15.92	< 0.001^*^

*ALT* alanine transaminase, U/L, *AST* aspartate aminotransferase, U/L, *γ-GT* γ-glutamyltransferase, U/L, *TBIL* total bilirubin, μmol/L, *DBIL* direct bilirubin, μmol/L, *TBA* total bile acid, μmol/L, *ALP* alkaline phosphatase, U/L, *PT* prothrombin time, s, *APTT* activated partial thromboplastin time, s, *INR* international normalized ratio, *Fib* ferritin, g/L, *Lac* lactate, mmol/L; Ammonia, μmol/L, *TNF-α* tumor necrosis factor α, pg/mL, *IL-6* interleukin 6, pg/mL, T0: baseline before treatment, T72: 72 h after treatment

^*^ indicates *P* < 0.05 compared with T0

### Independent factors for 28-day mortality of patients with sepsis-associated liver dysfunction

The clinical characteristics of patients with bacterial sepsis-associated liver dysfunction admitted to PICU were compared between survivors and non-survivors. In the present study, the characteristics of survivors and non-survivors were similar with respect to gender, PRISM III score, fever, complications, rate of hepatomegaly or hypoproteinemia, infection origin, infection markers (all *P* > 0.05) (Table 3). And there was no significant difference about 28-day mortality among difference bacterial aetiology (all *P* > 0.05), and there was no significant difference in respect of age (30 [2–132] months *vs.* 12 [2–192] months, *P* = 0.704) (Table 3). The ratio of continuous hemofiltration therapy was significantly higher in survivors than in non-survivors (78.6% *vs.* 38.5%, *P* < 0.05). Among the indicators for liver function, the levels of TBIL in non-survivors were significantly higher than that in survivors (232.99 ± 88.49 μmol/L *vs.*134.55 ± 127.51 μmol/L, *P* < 0.05) (Table 3). There were increasing trend but without statistical differences in respect of the levels of ALT, γ-GT, DBIL, TBA, INR, Lac, ammonia, TNF-α and IL-6 in non-survivors compared with survivors (all *P* > 0.05, Table 3). Multivariate logistic regression analysis showed that continuous hemofiltration therapy (Odd ratio [*OR*]: 0.091, 95% confidence interval [*CI*]: 0.009–0.948, *P* = 0.045) and the level of TBIL (*OR*: 20.980, 95% * CI*: 1.852–237.673, *P* = 0.014), but not age (*OR*: 0.578, 95% *CI*: 0.083–4.051, *P* = 0.578), were independent factors associated with 28-day mortality of patients with sepsis-associated liver dysfunction (Table 4). Furthermore, the continuous hemofiltration therapy and the levels of serum TBIL were significantly correlated with 28-day mortality of patients with bacterial sepsis complicated by liver dysfunction (*r* = − 0.408, *P* = 0.035; *r* = 0.42, *P* = 0.029, respectively).

**Table 3:** Comparison of biochemical and clinical parameters between survivor and non-survivor with sepsis-associated liver dysfunction initially infected by bacteria enrolled in study

Variables	Survivors (*n* = 14)	Non-survivors (*n* = 13)	*P*
Cases with CRRT, n (%)	11 (78.6%)	5 (38.5%)	0.034^*^
Age, month, median (range)	30 (2–132)	12 (2–192)	0.704
Male, n (%)	5 (35.7%)	8 (61.5%)	0.180
Stay in PICU, day, median (range)	9.5 (4–36)	10 (4–28)	1.000
Body temperature, ^o^C	39 (38–40)	38.8 (36–41)	0.678
Hepatomegaly, n (%)	10 (71.4%)	6 (46.2%)	0.182
Hypoproteinemia, n (%)	9 (64.3%)	13 (100%)	0.083
Pediatric Risk of Mortality III (PRISM III), median (range)	12.55 (10–17)	14.6 (10–20)	0.252
Co-organ dysfunction			
ARDS, n (%)	9 (64.3%)	4 (30.8%)	0.082
Kidney injury, n (%)	5 (35.7%)	6 (46.2%)	0.581
Encephalopathy, n (%)	4 (28.6%)	6 (46.2%)	0.585
Shock, n (%)	5 (35.7%)	8 (61.5%)	0.180
Infection origin			
Respiratory system, n (%)	8 (57.1%)	3 (23.1%)	0.072
Digestive system, n (%)	3 (21.4%)	4 (30.8%)	0.580
Urinary system, n (%)	2 (14.3%)	1 (7.7%)	0.586
Central nervous system, n (%)	1 (7.1%)	5 (38.5%)	0.050
Pathogens			
*Escherichia coli,* n	5 (35.7%)	5 (38.5%)	0.883
*Pseudomonas aeruginosa,* n	4 (28.6%)	2 (15.4%)	0.406
*Klebsiellapneumoniae,* n	1 (7.1%)	0 (0%)	1.000
*Streptococcus pneumoniae,* n	4 (28.6%)	3 (23.1%)	0.744
*Staphylococcus aureus,* n	0 (0%)	3 (23.1%)	0.172
Infection markers			
WBC	11.10 ± 8.48	12.96 ± 6.43	0.528
PCT	5.71 ± 3.64	6.13 ± 4.96	0.805
CRP	90.07 ± 44.65	106.92 ± 61.66	0.421
Liver function			
ALT	484.21 ± 128.89	643.94 ± 303.62	0.083
AST	1059.50 ± 848.05	954.94 ± 651.32	0.724
γ-GT	143.57 ± 69.92	205.46 ± 144.19	0.163
TBIL	134.55 ± 127.51	232.99 ± 88.49	0.029^*^
DBIL	108.89 ± 88.00	169.99 ± 79.64	0.071
TBA	92.22 ± 21.47	111.93 ± 31.51	0.067
ALP	166.43 ± 33.24	172.23 ± 32.84	0.653
PT	20.56 ± 6.47	31.62 ± 26.07	0.136
APTT	58.83 ± 23.16	66.46 ± 33.73	0.497
INR	1.55 ± 0.37	2.41 ± 1.94	0.117
Fib	1.51 ± 0.73	1.91 ± 0.76	0.171
Lac	4.20 ± 3.45	5.50 ± 3.99	0.410
Ammonia	139.07 ± 62.24	182.06 ± 75.11	0.122
TNF-α	96.89 ± 30.65	117.89 ± 37.66	0.123
IL-6	75.78 ± 21.75	92.27 ± 27.70	0.096

*ALT* alanine transaminase, U/L, *AST* aspartate aminotransferase, U/L, *γ-GT* γ-glutamyltransferase, U/L, *TBIL* total bilirubin, μmol/L, *DBIL* direct bilirubin, μmol/L, *TBA* total bile acid, μmol/L, *ALP* alkaline phosphatase, U/L, *PT* prothrombin time, s, *APTT* activated partial thromboplastin time, s, *INR* international normalized ratio, *Fib* ferritin, g/L, *Lac* lactate, mmol/L; Ammonia, μmol/L, *TNF-α* tumor necrosis factor α, pg/mL, *IL-6* interleukin 6, pg/mL

^*^ indicates *P* < 0.05 compared with survivors

**Table 4:** Multivariate logistic regression analysis of independent factors associated with 28-day mortality of patients with sepsis-associated liver dysfunction initially infected by bacteria

Variables	*OR*	95% *CI*	*P*
Continuous hemofiltration	0.091	0.009–0.948	0.045
TBIL	20.980	1.852–237.673	0.014
Age	0.578	0.083–4.051	0.578

*TBIL* total bilirubin, *OR* odd ratio, 95% CI 95% confidence interval

## Discussion

Continuous hemofiltration has been preferred as an effective adjuvant therapy for the treatment of systemic inflammatory syndromes in intensive care unit. In the present study, 28-day mortality of patients with bacterial sepsis-associated liver dysfunction was significantly reduced in the continuous hemofiltration group compared with the conventional management group, which was associated with decreasing the levels of TBIL, DBIL, TBA, Lac, TNF-α and IL-6 after 72 h treatment. And continuous hemofiltration therapy, as a protective factor, was significantly correlated with 28-day mortality of patients with bacterial sepsis complicated by liver dysfunction. To our knowledge, it is the first study to assess the clinical effect of continuous hemofiltration on 28-day mortality of bacterial sepsis-associated liver dysfunction in pediatric population.

The conventional management for liver dysfunction caused by bacteria includes antibiotics and supportive therapy to improve the disturbed homeostasis [18]. Despite the advances in conventional management, 28-day mortality of bacterial sepsis-associated liver dysfunction was 72.7% in our PICU. Though continuous hemofiltration is preferred to be effective tool to improve hemodynamics and fluid balance, there is controversial about whether continuous hemofiltration improves the mortality of critically ill. Previous study indicated that continuous hemofiltration application can significantly lower the 28-day mortality (38%* vs.* 71%, *P* = 0.011) and in-hospital mortality (62% *vs.* 86%, *P* = 0.04) in patients with severe burns and acute kidney injury [19]. However, the 72-h early-initiated continuous hemofiltration treatment has no effect on the 28-day mortality in patients with septic-shock-induced acute respiratory distress syndrome (ARDS) without acute kidney injury [20]. Our previous study indicated that continuous hemofiltration improved the inflammatory biomarkers but no advantage in mortality in patients with secondary hemophagocytic syndrome [21]. However, it is intriguing that continuous hemofiltration application significantly improved the 28-day mortality of patients with bacterial sepsis-associated liver dysfunction in our present study. Importantly, continuous hemofiltration therapy, as an independent protective factor, was significantly correlated with the prognosis of patients with bacterial sepsis complicated by liver dysfunction. Consistently, Deep et al. [12] reported that patients with pediatric acute liver failure (PALF) treated with continuous hemofiltration had a significantly increased chance of survival based on retrospective analysis (*HR*, 4; 95% *CI*, 1.5–11.6; *P* = 0.006). Otherwise, the survival rate of total 27 patients with bacterial sepsis-associated liver dysfunction was 51.85% (14/27) in our study. However, the survival rate of patient in continuous hemofiltration group was 68.7% in our study, which was similar to the 73.2% survival rate in patients with PALF in United states [22]. All these results indicated that continuous hemofiltration should be preferred effective adjuvant therapy to improve survival rate of patients with PALF and bacterial sepsis-associated liver dysfunction in children.

The timing to perform continuous hemofiltration is very important to improve the prognosis. The average interval time to initiate continuous hemofiltration after PICU admission was (22.06 ± 17.68) hours in our study. Consistently, Deep et al. reported that the average time to initiate continuous hemofiltration from PICU admission was (27 ± 6.9) hours [12]. Furthermore, we found that time to initiate continuous hemofiltration from PICU admission was significantly shorter in survivors compared with non-survivors, suggesting that the interval time between continuous hemofiltration initiation and PICU admission affects the outcome of bacterial sepsis-associated liver dysfunction. So, it is important to rapidly identify pathogen and indications for continuous hemofiltration initiation in patients with bacterial sepsis-associated liver dysfunction.

In the present study, continuous hemofiltration effectively decreased the levels of TBIL, DBIL, TBA, ammonia, Lac, TNF-α and IL-6. Our results are consistent with the report of previous study in acute liver failure before liver transplantation [21]. The levels of TBIL, DBIL, TBA, Lac, TNF-α and IL-6 were significantly decreased after 72 h treatment in the continuous hemofiltration group (all *P* < 0.05), other than in the conventional management group (all *P* > 0.05, Table 2). According to the introduction for Gambro prisma filter, molecules with molecular weight < 50 kDa could be wiped off. The bilirubin and lactate are “small” molecules, and TNF-α (~ 17 kDa–52 kDa peptide which circulates as a trimer) and IL-6 (~ 21 kDa) are “large” molecules. Generally, the more a molecule weighs, the larger it is in size and the more resistant it is to transport. So, the changes of serum TBIL, DBIL, TBA, Lac levels could be results from directly wiping off. However, continuous hemofiltration might regulate liver function as an adjuvant therapy. So, we speculated that the changes of serum TNF-α and IL-6 levels were contributed by both directly removing cytokines and indirectly regulating liver function. In addition, continuous hemofiltration can significantly improve the hemodynamic stability and neurological status in children with acute liver failure [23]. Whether hemodynamic stability and neurological status are influenced by continuous hemofiltration therapy in patients with bacterial sepsis-associated liver dysfunction should be investigated in the future.

Our study has some limitations. Firstly, this is a retrospective observational study with limited number of patients from a single center. This conclusion needs further study in prospective study with larger sample size. Secondly, some patients with severe coagulation disorders, or biofilm allergic reaction, or catheter difficult cannot perform continuous hemofiltration, although the patients were complicated with acute kidney injury, which would result in selection bias. Thirdly, we could not real-time monitor the changes of serum levels of TBIL, DBIL, TBA, Lac, ammonia, TNF-α and IL-6 during the treatment. Nevertheless, our results are noteworthy because continuous hemofiltration significantly improves 28-day mortality in pediatric patients with bacterial sepsis-associated liver dysfunction. More importantly, the present study brings an arousal of the potential benefits of continuous hemofiltration in pediatric patients with bacterial sepsis-associated liver dysfunction. It warrants further research using well-designed randomized controlled trials based on multi-site and larger simple size.

## Conclusions

In conclusion, 28-day mortality of patients with bacterial sepsis-associated liver dysfunction was significantly improved in the continuous hemofiltration group, which was related to the decreased serum levels of TBIL, DBIL, TBA, Lac, ammonia, TNF-α and IL-6 after 72 h treatment. Further larger simple size trial is needed to provide evidence on the role of continuous hemofiltration in bacterial sepsis-associated liver dysfunction.

## Additional file

Additional file 1: No. of patients, age, BMI, length of PICU stay, Body temperature, PRISM III, group (“0” for conventional group, “1” for CRRT group), prognosis (“0” for survivor, “1” for non-survivor), No. of organ dysfunction, co-morbidities (jaundice, hepatomegaly, shock, encephalopathy, renal injury, Hypoproteinemia, ARDS, Primary liver disease), site of infection, pathogen, biochemical indexes (CRP, PCT, WBC, ALT, AST, γ-GT, TBIL, DBIL, TBA, ALP, PT, APTT, Fib, INR, Lac, Ammonia, TNF-α and IL-6). The additional file includes the information about demographic and clinical characteristics of patients, group, co-morbidities, pathogen, and results of laboratory test before and after CRRT in patients with sepsis-associated liver dysfunction. (XLS 34 kb)

## Abbreviations

ACT: Activated coagulation time
ALT: Alanine aminotransferase
APTT: Activated partial thromboplastin time
DBIL: Direct bilirubin
G^−^: Gram-negative
G^+^: Gram-positive
IL-6: Interleukin − 6
INR: International normalized ratio
Lac: Lactate
LPS: Lipopolysaccharide
PICU: Pediatric intensive care unit
PRISM III: Pediatric Risk of Mortality III
PT: Prothrombin time
TBA: Total bile acids
TBIL: Total Bilirubin
TNF-α: Tumor necrosis factor-α
γ-GT: γ-glutamyltranspeptidase
